# Organizational characteristics of European pediatric onco-critical care: An international cross-sectional survey

**DOI:** 10.3389/fped.2022.1024273

**Published:** 2022-12-01

**Authors:** Jeppe S. A. Nielsen, Rachel Agbeko, Jessica Bate, Iolanda Jordan, Christian Dohna-Schwake, Jenny Potratz, Andrea Moscatelli, Gabriella Bottari, John Pappachan, Volker Witt, Roman Crazzolara, Angela Amigoni, Agniezka Mizia-Malarz, Mariá Sánchez Martín, Jef Willems, Marry M. van den Heuvel-Eibrink, Luregn J. Schlapbach, Roelie M. Wösten-van Asperen, the POKER (PICU Oncology Kids in Europe Research group) research consortium of ESPNIC (European Society of Paediatric & Neonatal Intensive Care), Joe Brierley

**Affiliations:** ^1^ Department of Neonatal and Pediatric Intensive Care, Rigshospitalet, Copenhagen, Denmark; ^2^ Department of Pediatric Intensive Care Unit, Great North Children's Hospital & Translational and Clinical Research Institute, Newcastle University, Newcastle Upon Tyne, United Kingdom; ^3^ Department of Pediatric Oncology, Southampton Children's Hospital, University Hospital Southampton NHS Foundation Trust, Southampton, United Kingdom; ^4^ Department of Pediatric Intensive Care, Hospital Sant Joan de Déu, University of Barcelona, Barcelona, Spain; ^5^ Department of Pediatric Intensive Care, University Hospital Essen, Essen, Germany; ^6^ Department of General Pediatrics-Intensive Care Medicine, University Children's Hospital Münster, Münster, Germany; ^7^ Department of Pediatric Intensive Care, Gaslini Hospital, Genova, Italy; ^8^ Department of Pediatric Intensive Care, Ospedale Pediatrico Bambino Gesù, IRCC, Rome, Italy; ^9^ Department of Pediatric Intensive Care, Southampton Children’s Hospital, Southamptom, United Kingdom; ^10^ Department of Pediatrics, St. Anna Children's Hospital, Medical University of Vienna, Southamptom, Austria; ^11^ Department of Pediatrics, Pediatric Intensive Care Unit, Medical University of Innsbruck, Innsbruck, Austria; ^12^ Department of Pediatric Intensive Care, Department of Woman's and Child's Health, Padua University Hospital, Padua, Italy; ^13^ Department of Pediatric Oncology, Hematology and Chemotherapy, Medical University of Silesia, Katowice, Poland; ^14^ Department of Pediatric Intensive Care, Hospital Universitario La Paz, Madrid, Spain; ^15^ Department of Pediatric Intensive Care, Ghent University Hospital, Ghent, Belgium; ^16^ Princess Máxima Center for Pediatric Oncology, Utrecht, Netherlands; ^17^ Department of Intensive Care and Neonatology, and Children's Research Centre, University Children's Hospital Zurich, Zurich, Switzerland; ^18^ Department of Pediatric Intensive Care, University Medical Centre Utrecht/Wilhelmina Children’s Hospital, Utrecht, Netherlands

**Keywords:** pediatric, intensive care, PICU, oncology, organization, structure

## Abstract

**Background:**

Intensified treatment protocols have improved survival of pediatric oncology patients. However, these treatment protocols are associated with increased treatment-related morbidity requiring admission to pediatric intensive care unit (PICU). We aimed to describe the organizational characteristics and processes of care for this patient group across PICUs in Europe.

**Methods:**

A web-based survey was sent to PICU directors or representative physicians between February and June 2021.

**Results:**

Responses were obtained from 77 PICUs of 12 European countries. Organizational characteristics were similar across the different countries of Europe. The median number of PICU beds was 12 (IQR 8–16). The majority of the PICUs was staffed by pediatric intensivists and had a 24/7 intensivist coverage. Most PICUs had a nurse-to-patient ratio of 1:1 or 1:2. The median numbers of yearly planned and unplanned PICU admissions of pediatric cancer patients were 20 (IQR 10–45) and 10 (IQR 10–30, respectively. Oncology specific practices within PICU were less common in participating centres. This included implementation of oncology protocols in PICU (30%), daily rounds of PICU physicians on the wards (13%), joint mortality and morbidity meetings or complex patients’ discussions (30% and 40%, respectively) and participation of parents during clinical rounds (40%).

**Conclusion:**

Our survey provides an overview on the delivery of critical care for oncology patients in PICU across European countries. Multidisciplinary care for these vulnerable and challenging patients remains complex and challenging. Future studies need to determine the effects of differences in PICU organization and processes of care on patients’ outcome.

## Introduction

Pediatric cancer patients admitted to a pediatric intensive care unit (PICU) form a unique patient population with specific critical care needs due to their underlying malignancy and treatment-related toxicities. Development of intensified and new treatment protocols have revolutionized oncology in the past decade and pediatric 5-year all-cancer survival currently stands at almost 80% (1). These treatment protocols are however, associated with severe side effects. Infections and treatment-related toxicity conditions are leading causes for of mortality and morbidity in cancer patients that require treatment in the intensive care unit and 2% to 28% of the pediatric cancer patients have been shown to require admission to the PICU during their disease course (2–8).

As cancer therapies improve and options evolve rapidly the knowledge, prompt recognition, and management of potentially life-threatening disease- and treatment-related complications is of utmost importance and requires close collaboration between the oncologists and PICU physicians. It has been shown in adult cancer patients that differences in ICU structure, organization, and collaboration between oncologists and the ICU team affects the quality of care and patient outcomes (9–11). The presence of clinical pharmacist in the ICU, presence of ICU protocols, and daily meetings between oncologists and intensivists were associated with lower hospital mortality even after adjustment for volume of exposure (11). In addition, implementation of protocols and daily meetings between ICU physicians and oncologists were also associated with more efficient ICU resource utilization. A survey among PICU and hematopoietic stem cell transplant (HSCT) physicians in 34 high-volume pediatric HSCT centers in the United States and Canada revealed significant variability on the clinical approach of critically ill HSCT patients (12). So far, no studies have addressed the organizational aspects of critical care for children with cancer. Comprehensive information on the organization of pediatric onco-critical care and the differences between PICUs is needed to further study the effects on outcomes, to harmonize care across units and to design future multicenter studies. To address these knowledge gaps, we aimed to describe the structure, organization, and delivery of critical care to children with cancer in Europe.

## Method

### Design and setting

Multinational survey initiated by the POKER consortium (Paediatric Oncology Kids in Europe Research group), endorsed by the European Society of Paediatric and Neonatal Intensive Care (ESPNIC).

We developed a web-based survey with domains based on prior studies demonstrating potential structure–outcome links in critical care and previously developed questionnaires (10, 11, 13, 14). The survey was constructed in accordance with the Checklist for Reporting Results of Internet E-Surveys (CHERRIES) (15). Details on the survey can be found in the Supplementary Material. The survey inquired practice outside the COVID-pandemic regulations. The full survey is shown in the Supplementary Material.

Between February and June 2021, the members of the POKER consortium representing 11 European countries approached PICU directors or representatives in their country by E-mail or *via* established networks and invited experienced colleagues to participate in the survey. To avoid duplicates only one representative for each PICU was contacted. The questionnaire was distributed online *via* SurveyMonkey. By partaking in the survey, participants consented to the use of their data for the purpose of the study.

### Data processing and statistical analysis

We screened data for duplicates, missing information, implausible and outlying values, and insufficient detail. In these cases, we contacted the local hospital representative to provide the requested additional information. Only completed questionnaires were analyzed. We grouped the participating countries as northern Europe (Denmark and Sweden), eastern Europe (Poland), central Europe (Austria, Belgium, Germany, France, Switzerland, the Netherlands), southern Europe (Italy, Spain) and the UK based on previous publications on European pediatric oncology patients (1).

Continuous variables were displayed as medians with interquartile ranges (IQRs). Categorical variables were displayed as frequencies (%). Due to the low numbers, no statistical comparisons were made.

## Results

### General hospital characteristics

A total of 226 surveys were sent out. Seventy-seven hospitals from 12 European countries, including the three independent pediatric cancer centers, completed the survey, resulting in a response rate of 34% (Supplementary Figure S1). We estimated a median response rate per country of 42% (IQR 31–50) (Supplementary Table S1). The median number of hospital beds was 118 (IQR 70–191) (Table 1). About half of the participants were from independent Children’s hospitals while the other half were co-located with adult hospitals. Seventy-five centers (97%) had their oncology ward and the PICU in the same hospital.

**Table 1:** General hospital characteristics.

Characteristic	All hospitals (*n* = 77)	Northern (*n* = 4)	Eastern (*n* = 11)	Central (*n* = 36)	Southern (*n* = 18)	UK (*n* = 8)
**Total beds**						
Median (IQR)	118 (70–191)	80 (45–86)	188 (78–290)	108 (70–191)	163 (70–218)	194 (131–290)
Independent Children’s hospital, *n* (%)*	38 (49)	1 (25)	5 (45)	22 (61)	7 (39)	3 (38)
**Oncology ward and PICU in same hospital, *n* (%)**						
Yes	75 (97)	4 (100)	11 (100)	35 (97)	17 (94)	8 (100)
If no, distance between hospitals				1.5 km	6.1 km	
**Annual newly diagnosed cancer patients**						
Median (IQR)	80 (41–120)	100 (68–100)	70 (43–90)	70 (45–106)	65 (29–135)	140 (135–225)
**Total oncology beds**						
Median (IQR)	17 (12–25)	22 (16–22)	32 (25–41)	15 (10–19)	16 (11–20)	20 (16–30)
**Total HSCT beds, *n* (%)**						
Median (IQR)	4 (0–24)	5 (3–7)	0 (0–5)	3 (0–6)	6 (2–6)	11 (7–12)

IQR, interquartile range; HSCT, hematopoietic stem cell transplantation; PICU, pediatric intensive care unit.

* Including the three independent pediatric cancer centers.

**Figure 1:**
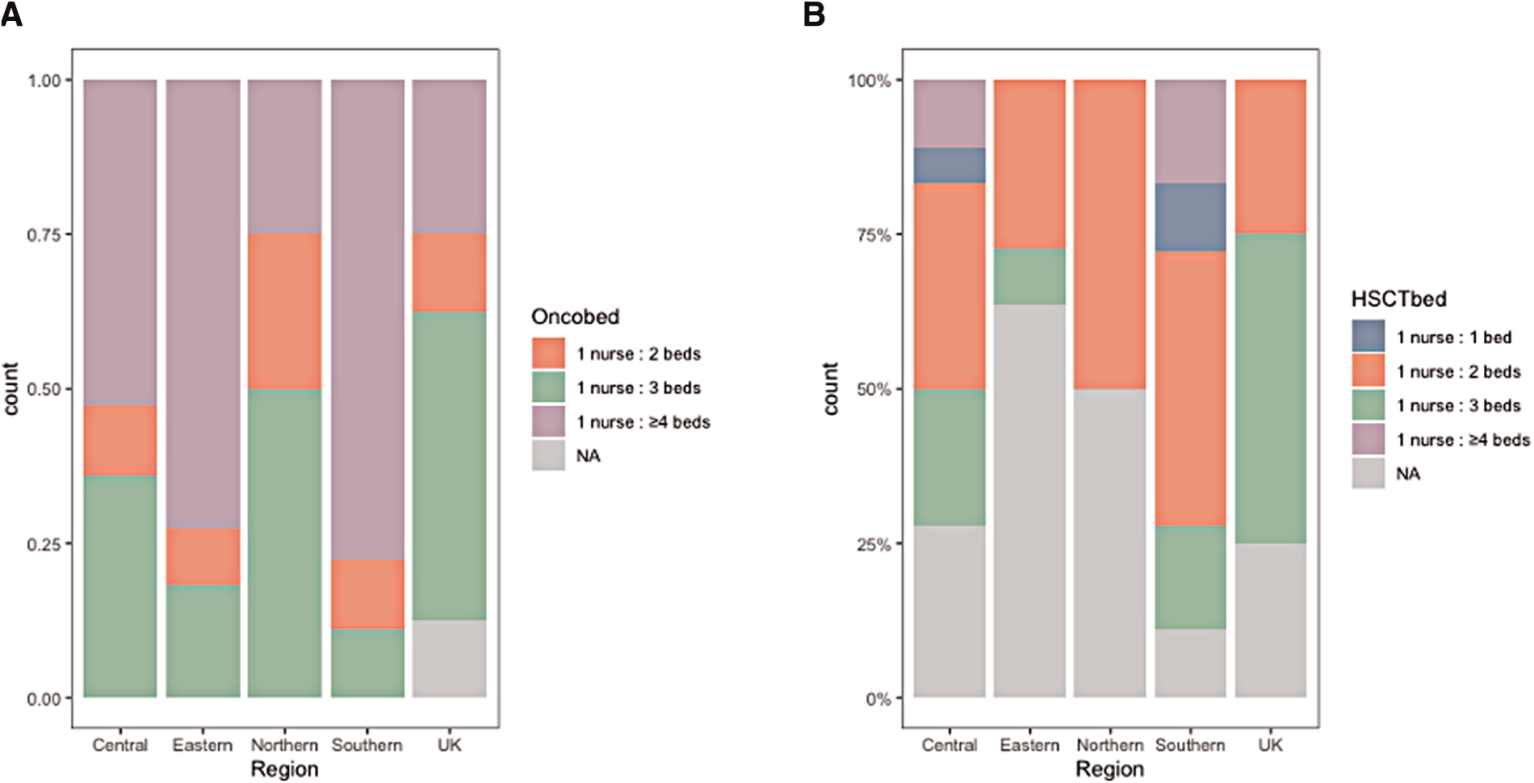
Nurse-to-bed ratio at the oncology ward (**A**) and HSCT ward (**B**) HSCT, hematopoietic stem cell transplantation.

The median number of newly diagnosed pediatric cancer patients per year was 80 (IQR 41–120) with a wide range from 5 to 1538. The median number of both oncology and hematopoietic stem cell transplant (HSCT) beds were similar among the different regions. Most centers had a 1:3 or 1:4 nurse-to-bed ratio for the oncology ward (Figure 1A). For the HSCT wards, the ratio was 1:2, except for the UK centers in which most centers had a ratio of 1:3 (Figure 1B).

### PICU organizational characteristics

The median number of PICU beds was 12 (IQR 8–16) (Table 2). Most of the participating units admitted a broad spectrum of patients, including medical (100%), surgical (97%), neurosurgical (86%), and trauma (90%) patients. Forty-nine% and 38% of the participating centers included cardiac surgical and burns patients, respectively.

**Table 2:** General characteristics of the PICUs.

Characteristic	All PICU (*n* = 77)	Northern (*n* = 4)	Eastern (*n* = 11)	Central (*n* = 36)	Southern (*n* = 18)	UK (*n* = 8)
**Total PICU beds**						
Median (IQR)	12 (8–16)	9 (5–15)	9 (13–16)	13 (8–16)	11 (7–16)	16 (14–19)
≤10	34	2	7	15	9	1
11–20	31	1	2	16	7	5
>20	12	1	2	5	2	2
**PICU patient population, *n* (%)**						
Medical	77 (100)	4 (100)	11 (100)	36 (100)	18 (100)	8 (100)
Surgical	75 (97)	4 (100)	10 (91)	35 (97)	18 (100)	8 (100)
Cardiac surgery	38 (49)	2 (50)	2 (18)	19 (53)	10 (56)	5 (28)
Neurosurgical	66 (86)	4 (100)	5 (45)	32 (89)	17 (94)	8 (100)
Trauma	69 (90)	4 (100)	9 (82)	31 (86)	17 (94)	8 (100)
Burn center	30 (39)	3 (75)	6 (55)	11 (31)	7 (39)	3 (38)
**Staffing, *n* (%)**						
Pediatric intensivists	68 (88)	2 (50)	8 (73)	36 (100)	14 (78)	8 (100)
Anesthetists	34 (44)	2 (50)	11 (100)	12 (33)	4 (22)	5 (63)
Pediatricians	29 (38)	1 (25)	5 (45)	20 (56)	2 (11)	1 (13)
Adult intensivists	8 (10)	2 (50)	3 (27)	2 (6)	1 (6)	0
Oncologists	11 (14)	1 (25)	3 (27)	6 (17)	1 (6)	0
PICU physician present in PICU 24/7, *n* (%)	71 (92)	4 (100)	10 (91)	31 (86)	18 (100)	8 (100)
**Annual PICU admissions**						
Total, Median (IQR)	450 (290–750)	300 (165–325)	150 (110–236)	550 (350–950)	425 (305–660)	755 (689–895)
**Annual oncology and HSCT PICU admissions**						
Planned Oncology						
Median (IQR)	20 (10–45)	35 (17–53)	8 (2–11)	28 (10–43)	30 (15–45)	25 (20–38)
**Unplanned Oncology**						
Median (IQR)	10 (10–30)	25 (15–38)	7 (5–13)	18 (10–30)	18 (10–34)	25 (14–48)
Planned HSCT	0 (0–2)	0 (0–4)	0 (0–0)	0 (0–0)	2 (0–5)	1 (0–7)
Median (IQR)						
Unplanned HSCT	3 (0–7)	5 (0–11)	0 (0–4)	2 (0–5)	6 (2–10)	7 (3–12)
Median (IQR)						
**Technologies available besides MV and vaso-active support, *n* (%)**						
Hemodialysis and/or CRRT	69 (90)	3 (75)	9 (82)	31 (86)	18 (100)	8 (100)
Plasmapheresis or plasma exchange	65 (84)	3 (75)	8 (73)	29 (81)	17 (94)	8 (100)
ECMO	41 (53)	2 (50)	3 (27)	21 (58)	11 (61)	4 (50)
None of the above	8 (10)	1 (25)	2 (18)	5 (14)	0	0
**Isolation possibilities, *n* (%)**						
Geographic isolation	62 (81)	3 (75)	8 (73)	32 (89)	12 (67)	7 (88)
High-efficiency air filtration	45 (58)	3 (75)	7 (64)	18 (50)	10 (56)	7 (88)
No isolation	3 (4)	0	1 (9)	1 (3)	1 (6)	0

CRRT, continuous renal replacement therapy; ECMO, extracorporeal membrane oxygenation; HSCT, hematopoietic stem cell transplantation; IQR, interquartile range; PICU, pediatric intensive care unit.

**Figure 2:**
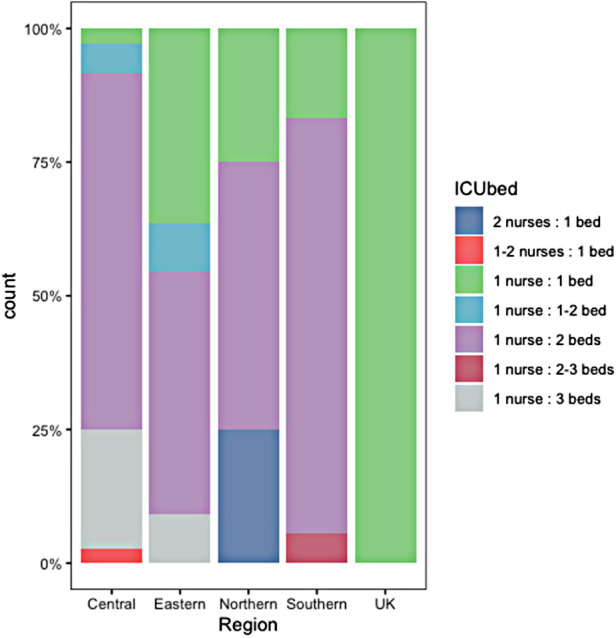
PICU nurse-to-bed ratio. PICU, pediatric intensive care unit.

Sixty-eight PICUs (88%) were staffed by pediatric intensivists. In some centers anesthetists (*n* = 34, 44%), general pediatricians (*n* = 29, 38%, especially in eastern and central European PICUs), adult intensivists (*n* = 8, 10%), and oncologists (*n* = 11, 14%) were part of the PICU team. Six PICUs (8%) did not have a 24/7 intensivist coverage. Sixty-six PICUs (86%) had a 1:1 or 1:2 nurse-to-bed ratio (Figure 2).

The median number of PICU admissions per year was 450 (IQR 290–750) with a range of 10 to 2800 admissions per year. PICU admissions per region were 300 (IQR 165–325) in northern Europe, 150 (IQR 110–236) in eastern Europe, 550 (IQR 350–950) in central Europe, 425 (IQR 305–660) in southern Europe, and 755 (IQR 689–895) in the UK. The median numbers of PICU admissions of pediatric cancer and HSCT patients were similar across the European regions with 20 (IQR 10–45) planned and 10 (IQR 10–30) unplanned admissions of cancer patients. Numbers of HSCT patients admitted to PICU were lower [median 3 (IQR 0–7)] compared to number of oncology patients and were predominantly unplanned.

Sixty-nine centers (90%) offered hemodialysis, plasmapheresis, or plasma exchange (*n* = 65, 84%) while 41 centers (53%) had in-house extracorporeal membrane oxygenation (ECMO) programs. Most of the participating centers had isolation capacity, either in the form of geographic isolation (81%) or patient rooms with high-efficiency air filtration (58%). Of note, 4% of the participating centers had no isolation facility in their PICU.

### Care for the critically ill pediatric oncology patients

Seventy-five centers completed the questions on care characteristics for critically ill pediatric oncology patients. Two centers from Central Europe provided only information on general hospital and PICU organizational characteristics. Sixty-eight centers (91%) had general PICU protocols for sepsis and infection prevention, and 51 centers (68%) had strategies for ventilation (Table 3). However, only 25 centers (33%) had specific PICU protocols for patients with cancer. Administration of chemotherapy during critical illness was possible in almost all centers (99%).

**Table 3:** Care for the critically ill pediatric oncology patient.

Characteristic	All hospitals (*n* = 75)	Northern (*n* = 4)	Eastern (*n* = 11)	Central (*n* = 34)	Southern (*n* = 18)	UK (*n* = 8)
**General PICU Protocols, *n* (%)**						
VAP prevention	45 (60)	2 (50)	7 (64)	16 (47)	14 (78)	6 (75)
Central line–associated bloodstream	68 (91)	3 (75)	10 (91)	30 (88)	17 (94)	8 (100)
Infection prevention						
Urine tract-infection prevention	38 (51)	1 (25)	10 (91)	13 (38)	12 (67)	2 (25)
Sepsis management	68 (91)	4 (100)	10 (91)	30 (88)	16 (89)	8 (100)
Weaning from MV	41 (55)	2 (50)	6 (55)	19 (56)	10 (56)	4 (50)
Lung protective ventilation	51 (68)	2 (50)	6 (55)	27 (79)	11 (61)	5 (63)
Non-invasive ventilation protocol	51 (68)	3 (75)	8 (73)	21 (62)	13 (72)	6 (75)
Sedation in patients with MV	59 (79)	2 (50)	7 (64)	28 (82)	15 (83)	7 (88)
Early mobilization	36 (48)	3 (75)	7 (64)	17 (50)	4 (22)	5 (63)
Nutritional protocols	64 (85)	4 (100)	11 (100)	26 (76)	16 (89)	7 (88)
Antimicrobial stewardship program	58 (77)	4 (100)	7 (64)	22 (65)	17 (94)	8 (100)
**Specific Oncology PICU protocols, *n* (%)**						
Febrile neutropenia	25 (33)	1 (25)	4 (36)	11 (32)	5 (28)	4 (50)
Invasive fungal infections	19 (25)	1 (25)	3 (27)	10 (29)	3 (17)	2 (25)
Tumor lysis syndrome	24 (32)	0	4 (36)	12 (35)	5 (28)	3 (38)
Chemotherapy at the PICU	18 (24)	0	3 (27)	9 (26)	3 (17)	3 (38)
Other^8^	4 (5)	0	1 (9)	1 (3)	2 (11)	1 (13)
Chemotherapy at the PICU, *n* (%)	74 (99)	4 (100)	10 (91)	34 (100)	18 (100)	8 (100)
PEWS, *n* (%)	31 (41)	2 (50)	6 (55)	6 (18)	9 (50)	8 (100)
Daily rounds PICU physicians at the oncology and HSCT wards, *n* (%)	10 (13)	1 (25)	0	5 (15)	4 (22)	0
Daily rounds of the oncologists at the PICU, *n* (%)	70 (93)	4 (100)	8 (73)	34 (100)	16 (89)	8 (100)
**Discussions, *n* (%)**						
Mortality discussions	23 (31)	1 (25)	3 (27)	8 (24)	7 (39)	4 (50)
Complication discussions	29 (39)	1 (25)	4 (36)	10 (29)	9 (50)	5 (63)
Complex-patient discussions	37 (49)	2 (50)	3 (27)	14 (41)	13 (72)	5 (63)
Rapid response team, *n* (%)	58 (77)	3 (75)	11 (100)	27 (79)	11 (61)	6 (75)
Cardiac-arrest team, *n* (%)	62 (83)	4 (100)	9 (82)	30 (88)	12 (67)	7 (88)
**Respiratory support at the oncology/HSCT ward, *n* (%)**						
Yes	28 (37)	3 (75)	2 (18)	9 (26)	10 (56)	4 (50)
HFNC	27 (36)	3 (75)	1 (9)	9 (26)	10 (56)	4 (50)
NIV CPAP/BiPAP	6 (8)	2 (50)	0	1 (3)	3 (17)	0
Established long term ventilation *via* tracheostomy	7 (9)	1 (25)	1 (9)	1 (3)	4 (22)	0
Vaso-active support at the oncology/HSCT ward, *n* (%)	10 (13)	1 (25)	0	4 (12)	5 (28)	0
Renal replacement therapy at the oncology/HSCT ward, *n* (%)	13 (17)	1 (25)	5 (45)	3 (9)	2 (11)	1 (13)
Palliation service, *n* (%)	64 (85)	3 (75)	8 (73)	31 (91)	15 (83)	7 (88)
Pain management service, *n* (%)	70 (93)	4 (100)	11 (100)	33 (97)	14 (78)	8 (100)
Psychological service, *n* (%)	74 (99)	4 (100)	11 (100)	34 (100)	17 (94)	8 (100)
**Change of goals of care, *n* (%)**						
Both at the PICU and ward	48 (64)	1 (25)	6 (55)	21 (62)	14 (78)	6 (75)
At the ward	20 (27)	3 (75)	4 (36)	10 (29)	2 (11)	1 (13)
At the PICU	5 (7)	1 (25)	0	3 (9)	1 (6)	1 (13)
Outpatient clinic	2 (3)	1 (25)	1 (9)	1 (3)	1 (6)	0
**PICU consultants involved in goals of care discussion, *n* (%)**						
Yes	45 (60)	1 (25)	4 (36)	21 (62)	14 (78)	5 (63)
No	4 (5)	0	1 (9)	2 (6)	1 (6)	0
Sometimes	26 (35)	3 (75)	6 (55)	11 (32)	3 (17)	3 (37)

BiPAP, bilevel positive airway pressure; CPAP, continuous positive airway pressure; HFNC, high flow nasal cannula; HSCT, hematopoietic stem cell transplantation; MV, mechanical ventilation; NIV, non-invasive ventilation; PEWS, pediatric early warning score; PICU, pediatric intensive care unit; VAP, ventilator associated pneumoniae.

CAR-T cell protocol, Cytokine release syndrome, Antimicrobial therapy specific for oncology and HSCT patients, PICU admission criteria.

Overall, 41% of the centers had implemented a Pediatric Early Warning Score (PEWS), whereas in the UK all centers used a PEWS. In 10 centers (13%) there were daily rounds of PICU physicians on the oncology and HSCT wards. In contrast, in 70 centers (93%) there were daily rounds of oncologists in the PICU when oncology patients were admitted. Twenty-three centers (31%) hold joint oncology and intensivist mortality meetings, 29 centers (39%) joint complication meetings, and 37 centers (49%) hold joint complex-patient discussions. Most of the centers had a rapid response team or a cardiac arrest team, 77% and 83% respectively.

One-third of the oncology units provided organ support on the ward. Twenty-seven centers (36%) offered high-flow nasal cannula, six centers (8%) non-invasive continuous positive airway pressure/bilevel positive airway pressure (NIV CPAP/BiPAP), and seven centers (9%) established long-term ventilation on tracheotomies. Inotropic support and renal replacement therapy were possible in 10 (13%) and 13 (17%) centers, respectively.

Palliation, pain, and psychological services to patients and families, were available at almost all hospitals, however psychology service for staff was only available in 63% of the participating units. Changes in goals of care towards palliation mainly took place both at the oncology ward and PICU in 48 center (64%). In 45 centers (60%) PICU consultants were involved in these discussions.

### Care for the parents and family members at the PICU

Visiting hours on the PICU was usually 24 h per day. However, some eastern European units only allowed 2 (IQR 2–10) h of visits per day (Table 4). The median number of family members allowed was two and about 50% of the PICUs have the possibility of rooming in for the parents. Information to caretakers was often given by multidisciplinary team (60%) and separate meeting rooms were available in 79% of the hospitals.

**Table 4:** Care for the parents and family members at the PICU.

Characteristic	All hospitals (*n* = 75)	Northern (*n* = 4)	Eastern (*n* = 11)	Central (*n* = 34)	Southern (*n* = 18)	UK (*n* = 8)
**Visiting hours**						
Median (IQR)	24 (12–24)	24 (24–24)	2 (2–10)	24 (22–24)	24 (9–24)	24 (24–24)
**No. of parents/family members allowed**						
Median (IQR)	2 (1–2)	2 (2–4)	1 (1–2)	2 (2–2)	2 (1–2)	2 (2–2)
Rooming-in possibility, *n* (%)	35 (47)	4 (100)	1 (9)	19 (56)	7 (39)	4 (50)
Room for family conferences, *n* (%)	59 (79)	4 (100)	6 (55)	27 (79)	14 (78)	8 (100)
Participation parents in patient care, *n* (%)	66 (88)	4 (100)	3 (27)	34 (100)	17 (94)	8 (100)
Participation parents in clinical rounds, *n* (%)	33 (44)	4 (100)	1 (9)	17 (50)	5 (28)	6 (75)

IQR, interquartile range.

The parents were mostly allowed to participate in patient care (88%), while 44% of units allow parents to join clinical rounds.

## Discussion

This multicenter survey was performed to assess the characteristics of critical care organization for children with cancer across Europe. The general PICU organization appeared fairly comparable among the participating countries with the 24/7 presence of an intensivist, use of general PICU protocols, nurse-to-bed ratio, and available PICU resources. Half of the participating centers had ECMO facilities. Almost all centers were able to administer chemotherapy in PICU and had daily rounds of the oncologists at the PICU. However, a low number of centers has oncology specific PICU protocols, joint mortality-morbidity and complex-case discussions, and participation of parents in daily rounds.

ICU size was equally distributed within the total group, with a median of 12 beds per PICU. Albeit the range was from 3 to 50 beds, this is comparable with previous studies carried out in Europe and the USA (10, 13, 14, 16). The total number of annual PICU admissions differed more between the different countries varying from 150 admissions in Poland to 755 in the UK. At the same time, annual PICU admissions of oncology and HSCT patients were similar between the different regions.

Patients with cancer represent among the most complex patient populations in medicine (17), and acute critical illness adds additional complexity. Given the improving survival rates alongside advances in therapeutic options, more pediatric cancer patients are expected to require advanced life support for cancer-related complications, treatment-related toxicities, and severe infections. Specialization in other critical care areas such cardiac ICU (CICU) has been well established and recognized followed by improved outcome. Therefore, development of dedicated oncological PICUs or further specialization in critical care oncology may need to be explored. So far, no studies have enlightened the effect of differences in organizational structure and processes of care, hospital and PICU case volume, multidisciplinary approach, availability of supporting services such palliative care services on pediatric cancer patient outcomes.

In 71% of the participating PICUs in our survey, there were 24/7 in-house intensivists, which is comparable to a previous European PICU survey in 2000 (14). There are multiple studies showing improved outcomes with 24 h in-hospital pediatric critical care physician (18, 19). In some countries, 50% of the PICUs medical staff was not pediatric intensivists. This could partly be explained by some management traditions i.e. anesthesiologist leading ICUs or joint NICU/PICUs or adult ICU/PICU (20). In our survey, the nurse-to-bed ratio in most PICUs was 1:1 or 1:2, which is in line with the ratios found in a large survey among PICUs in the USA (14). In adults, it has been shown that a higher nurse staffing was associated with improved survival (21). An increase of the nurse-to-bed ratio from 1:2 to 1:1.5 was associated with a 1.8% decrease in mortality. Currently, no data are available on associations of nurse-to-bed-ratio with survival of pediatric ICU patients.

As has been shown in adult cancer patients, close collaboration between oncologists and intensivists for care planning and the joint setting of daily goals were independently associated with lower hospital mortality and more efficient ICU resource use (11). In our survey, daily rounds of the oncologist at the PICU were documented in 93% of the participating centers. Setting or changing goals of care took place both at the PICU and the ward in almost 2/3 of the centers, and often the PICU consultants were involved in these goals of care discussions. The co-location of the PICU and oncology wards in 97% of the participating centers may have facilitated communication amongst the PICU physicians and oncologists. Our results are in line with results from a 2011 North American survey among pediatric intensive care and HSCT physicians on the care of critically ill children after HSCT which also showed variability in practice (12).

Nowadays, participation of parents in daily rounds is advocated. Parental involvement in multidisciplinary rounds in pediatrics is associated with shortened stays, earlier discharges, reduced costs, and improved provider satisfaction (22, 23). In 43% centers of the participating centers, parents participate in clinical rounds. Implementing standardized process for multidisciplinary rounds, including the presence of parents, may improve communication amongst the healthcare team, facilitate dialogue between patients’ families and the healthcare team, and reduce safety events (24).

Co-location of oncology ward and PICU also allows for timely review of clinically deteriorating patients. The outcome of critically ill patients with cancer is in part determined by timely recognition of clinical deterioration and the treatment they received before their PICU admission. Physicians and nurses who take care of cancer patients should therefore be skilled at detecting warning signs of clinical deterioration and be familiar with the essential therapeutic measures needed. Medical emergency teams that are staffed by members of the critical care team may support the teams on the ward with identifying and managing deteriorating patients and may facilitate the transition to PICU. Rapid response teams and cardiac arrest teams were present in 75%–80% of the hospitals—both mostly occupied with pediatric intensivists (60%) and otherwise staffed with anesthetists, adult intensivist, senior or junior pediatricians, and PICU nurses. In 10 centers there were daily rounds of PICU physicians at the oncology and HSCT wards. Surprisingly, only 40% of the participating centers were using an early warning score. There is some evidence showing significant benefits from PEWS on patients’ outcome while others fail to depict the same beneficial outcome benefits (25–28). However, a recent systematic review shows that there is still a gap of knowledge in both predictive performance and impact of PEWS in the high-risk population of pediatric oncology patients (29).

Palliative care is a key component of comprehensive care for patients with cancer and should be an essential collaborator to pediatric oncology PICU care (30). Eighty-five percent of the centers have a palliation service. Psychology service for patients and/or parents was available in 98% of the participating centers. However, in only 63% of centers, psychology service was available for the staff members. One aspect of critical care for patients with cancer that is often overlooked is the impact on the health care providers taking care of the patients with cancer. The ICU environment is stressful not only for patients but also for the ICU staff. Caring for patients with cancer often presents critical care teams with unique medical and ethical challenges that can lead to conflict, moral distress, and burnout (31–33). Perceived inappropriateness of ICU care can cause job dissatisfaction in ICU nurses and physicians (34).

Our study has several important limitations. Due to incomplete response from PICU units across Europe and the survey originating in the POKER network, a higher percentage of participation in hospitals with pediatric oncology could potentially induce selection bias. There is also risk of less participation from minor units as not all pediatric intensivists are ESPNIC members or receive ESPNIC correspondence (35). For example, Poland was the only country from eastern Europe participating in the survey. As we included 77 units out of a total of 226 PICUs in the participating European countries, it is highly likely that this may limit the external validity of this study. Finally, we surveyed general characteristics of onco-critical care, but we did not assess severity of illness scores, patient outcomes, or resource use in the PICU. We acknowledge that in-depth information is needed to determine whether differences in care are associated with short and long-term outcomes, and more efficient resource utilization. Further analysis of key factors in structure and organization may thus help us to improve overall quality of care for the oncology patients in the pediatric ICU.

This is the first cross-sectional study depicting size, workflow, attending staff, service provisions, and resources of European PICUs with focus on patients with underlying malignancies. Albeit size, staffing and service provisions seems comparable there is also variation, especially regarding multidisciplinary care. In addition to providing optimal care to critically ill patients, multidisciplinary teams offer the ideal platform to perform multidisciplinary research which is required to achieve significant improvements in the care of critically ill pediatric patients with cancer (36). Future studies should address severity illness across European PICUs to determine baseline comparability and the effect of the differences found in the delivery of care on patient outcomes and ICU resource use.

## Data Availability

The datasets generated for this study are available on request to the corresponding author.

## POKER consortium collaborators

Joe Brierley, Department of Pediatric Intensive Care, Great Ormond Street Hospital NHS Foundation Trust, London, UK; Simon Nadel, Department of Pediatric Intensive Care, Imperial College London, London, UK; Omer Aziz, Department of Pediatric Intensive Care, Bristol Royal Hospital for Children, Bristol, UK; Mark Worrall and Diana McIntosh, Royal Hospital for Children, Glasgow, UK; Simon Vieth, Department of Pediatrics, University Medical Center Schleswig-Holstein, Kiel, Germany; Eva Tschiedel, University Hospital Essen, Essen, Germany; Frédéric Valla and Solenn Remy, Department of Pediatric Intensive Care, Centre Hospitalier Universitaire Lyon, Lyon-Bron, France; Frida Rizzati, Department of Pediatric Hematology-Oncology Unit, Division of Pediatrics, Woman-Mother-Child Department, Lausanne University Hospital (CHUV) and University of Lausanne (UNIL), Lausanne, Switzerland; Andrea Titieni, Department of Pediatric Intensive Care, St. Anna, Vienna, Austria; Emanuele Rossetti, Department of Pediatric Intensive Care, Bambino Gesù Children’s Hospital, Rome, Italy; Fabio Caramelli and Maria Cristina Mondardini, Department of Pediatric Intensive Care, S. Orsola-Malpighi University Hospital, Bologna, Italy; Alessia Montaguti, Department of Pediatric Intensive Care, Gaslini Hospital, Genova, Italy.
