# Supplementary material for: Organizational characteristics of European pediatric onco-critical care: An international cross-sectional survey
Source: Front Pediatr. 2022 Dec 1;10:1024273. doi: 10.3389/fped.2022.1024273 (PMC9751627; doi:10.3389/fped.2022.1024273)
Supplement: Supplementary file 1 [file Datasheet1.docx]

**Supplementary material**

**Content**

| Web based survey | Page 2-7 |
| --- | --- |
| Figure S1 | Page 8 |
| Table S1 | Page 9 |
|  |  |

**Development of the web-based survey**

We developed a web-based survey with domains based on prior studies demonstrating potential structure–outcome links in critical care and previously developed questionnaires. Additional questions were designed by the POKER consortium.

The first draft of the questionnaire was discussed in January 2021. The POKER network is a pan-European network with board members across most European countries who have all endorsed the questionnaire. The survey was reviewed and piloted by the board members from different European countries on interpretation, content, usability, and technical functionality of the electronic questionnaire. The survey was revised following feedback from members before distribution to the European centers. The survey was not translated into other languages so there may have been some misinterpretation of questions. However, data on relevant outliers were validated by contact to local investigators and corrected if necessary. After several rounds of review and piloting by the POKER consortium, the final survey included the following domains: hospital and PICU type and bed capacity, PICU staffing patterns, hospital policies on the use of early warning scores and rapid response teams, presence of regular multidisciplinary clinical rounds, clinical pathways and protocols, regular meetings between oncologists and intensivists (i.e. formal meetings between the attending oncologist and intensivist to discuss care planning and setting of goals), oncologist participation during PICU multidisciplinary rounds, PICU admission policies for children with cancer, regular debriefing and administrative multidisciplinary meetings, and family visiting policies. The mean time spent completing the survey was 12min:35sec.

**Organizational Characteristics of European Onco-Critical Care.**

**Thank you for participating in our survey. Your feedback is important.**

**It is imperative to differentiate what defines a PICU patient in the different European countries for future projects with comparisons across European Pediatric Intensive Care Units. This is especially true regarding pediatric oncology patients' complex care where different countries and/or hospitals can have different approaches and capabilities. Therefore, PICU outcomes will depend on an accurate description of these circumstances. This questionnaire will therefore elaborate on the differences between European PICUs with a special focus on oncology patients.**

**Participation in this study will be limited to this one-time survey. The survey should not take any longer than 20 minutes of your time.**

**Please click the link below and fill out the following survey. Your completion of the survey implies your voluntary consent to participate in the research.**

**Thank you in advance for your time!**

**All questions relate to the pre-COVID situation!**

|  |  |
| --- | --- |
| Country | - Austria - Belgium - Denmark - France - Germany - Italy - Poland - Spain - Switzerland - The Netherlands - UK - …. |
| Site Name |  |
| **Hospital and PICU characteristics** | |
| Independent Children’s hospital | - Yes - No |
| Number of general **pediatric, non-PICU** hospital beds | …………………. |
| Oncology ward and PICU in the same hospital | - Yes - No |
| If no, distance between hospitals | …………………. (kilometers) |
| Number of active **total** PICU beds in your hospital | …………………. |
| PICU patient population | - Medical - Surgical - Cardiac Surgical - Neurosurgical - Trauma - Oncology - Stem Cell Transplant - Solid Organ Transplant - Burn Center   (Types of patients seen in your PICU (choose all that apply) |
| Estimated annual **total** PICU admissions | ………………….  (Approximate number of annual PICU admissions in your PICU) |
| Estimated annual PICU admissions of **planned oncology** patients (e.g. post-neurosurgery) | ………………….  (Approximate number of annual PICU admissions in your PICU) |
| Estimated annual PICU admissions of **unplanned oncology** patients (e.g. sepsis, acute intracranial hypertension) | ………………….  (Approximate number of annual PICU admissions in your PICU) |
| Estimated annual PICU admissions of **planned HSCT** patients | ………………….  (Approximate number of annual PICU admissions in your PICU) |
| Estimated annual PICU admissions of **unplanned HSCT** patients | ………………….  (Approximate number of annual PICU admissions in your PICU) |
| Average overall bed/ICU nurse ratio | - 1 nurse : 1 bed - 1 nurse : 2 beds - 1 nurse : 3 beds - 1 nurse : 4 beds - Other (please specify) |
| Consultants working in PICU | - Pediatric intensivists - Anesthetists - Pediatricians - Adult intensivists - Oncologists   (Types of physicians in your PICU (choose all that apply) |
| Technologies available in your PICU | - Hemodialysis and/or continuous renal replacement therapy - Plasmapheresis or plasma exchange - Extracorporeal membrane oxygenation - None of the above   (Types of technologies used in your PICU (choose all that apply) |
| If technologies are not available, will the patient be transferred to another hospital? | - Yes - No |
| Isolation possibilities at your PICU | - Geographic isolation (separate patient’s room) - Patient’s room with high-efficiency air filtration - No isolation possibilities available |
| **Characteristics of oncology and HSCT ward** | |
| Number of newly diagnosed cancer patients/year | …………………. |
| Is the HSCT unit integrated in the oncology unit? | - Yes - No |
| Number of beds on oncology ward | …………………. |
| Number of beds on HSCT ward | …………………. |
| Nurse/bed ratio on oncology ward (daytime) | - 1 nurse : 1 bed - 1 nurse : 2 beds - 1 nurse : 3 beds - 1 nurse : 4 beds - Other (please specify) |
| Nurse/bed ratio on HSCT unit (daytime) | - 1 nurse : 1 bed - 1 nurse : 2 beds - 1 nurse : 3 beds - 1 nurse : 4 beds - Other (please specify) |
| **Process of care** | |
| In your unit, do you have a protocol on xy (list below) in place? | - VAP prevention - Central line–associated bloodstream infection prevention - Urine tract-infection prevention - Sepsis management - Weaning from MV - Lung protective ventilation - Non-invasive ventilation protocol. - Sedation in patients with MV - Early mobilization - Nutritional protocols - Antimicrobial stewardship program - Not applicable   (Choose all that apply) |
| Do you have specific clinical protocols for patients with cancer in PICU? | - Yes - No |
| If yes, type of protocol | - Febrile neutropenia - Invasive fungal infections - Tumor lysis syndrome - Chemotherapy at the PICU - Other, please specify….   (Choose all that apply) |
| Is there a possibility to administer chemotherapy at the PICU during critical illness? | - Yes - No |
| **Family care and ICU visiting policies** | |
| Visiting hours per day | …………………. |
| Allowed No. of parents/family members at bedside | …………………. |
| Possibility for rooming-in for parents | - Yes - No |
| Specific room for family conferences | - Yes - No |
| Provision of booklet/electronical information/app with general information on PICU organization and policies | - Yes - No |
| Who provides **daily** patient information provided to the family? | - PICU consultant - Jointly by multidisciplinary team (PICU consultant, oncologist, surgeon, if applicable) |
| Who provides **Important** patient information to the family? | - PICU consultant - Jointly by multidisciplinary team (PICU consultant, oncologist, surgeon, if applicable) |
| Parents can participate in clinical rounds (e.g. participation in daily rounds, multidisciplinary discussions) | - Yes - No |
| Parents can participate in patient care (e.g. washing, changing dipers) | - Yes - No |
| **Early recognition of clinical deterioration at the ward** | |
| Does your hospital use a Pediatric Early Warning Score or other warning score? | - Yes - No |
| Does your hospital have a 24/7 in-hospital PICU physician? | - No - Resident - PICU fellow - Pediatric intensivist - Pediatric intensivist or PICU fellow   (Choose all that apply) |
| Does your hospital have a rapid response team? | - Yes - No |
| If yes, who participates in the rapid response team? | - Pediatric intensivist - Anesthetists - Adult intensivist - Senior pediatrician - Junior pediatrician - PICU nurse - Other, specify …..   (Choose all that apply) |
| Does your hospital have a cardio-arrest team? | - Yes - No |
| If yes, who participates in the cardio-arrest team? | - Pediatric intensivist - Anesthetists - Adult intensivist - Senior pediatrician - Junior pediatrician - PICU nurse - Other, specify….   (Choose all that apply) |
| Does your oncology ward deliver respiratory support apart from low flow oxygen? | - Yes - No |
| If yes, | - High flow Nasal Cannula - NIV CPAP - NIV BiPAP - Established long term ventilation via tracheostomy   (Choose all that apply) |
| Does your HSCT ward deliver respiratory support apart from low flow oxygen? | - Yes - No |
| If yes, | - High flow Nasal Cannula - NIV CPAP - NIV BiPAP - Established long term ventilation via tracheostomy   (Choose all that apply) |
| Does your oncology ward deliver inotrope/vasopressor treatment? | - Yes - No |
| If yes, | - Dopamine - Dobutamine - Epinephrine - Norepinephrine - Milrinone - Levosimendan   (Choose all that apply) |
| Does your HSCT ward deliver inotrope/vasopressor treatment? | - Yes - No |
| If yes, | - Dopamine - Dobutamine - Epinephrine - Norepinephrine - Milrinone - Levosimendan   (Choose all that apply) |
| Does your oncology ward deliver renal replacement therapy? | - Yes - No |
| If yes, | - Continuous hemodialysis - Continuous hemofiltration - Continuous hemodiafiltration - Intermittent renal replacement therapy - Peritoneal dialysis - (Choose all that apply) |
| Does your HSCT ward deliver renal replacement therapy? | - Yes - No |
| If yes, | - Continuous hemodialysis - Continuous hemofiltration - Continuous hemodiafiltration - Intermittent renal replacement therapy - Peritoneal dialysis   (Choose all that apply) |
| Does the hospital have high dependency unit (HDU-unit) outside the PICU where more intensive therapy (respiratory, cardiac or RRT or other) can be supported? | - Yes - No |
| **Clinical decision making for patient care** | |
| Are there daily rounds of the PICU physicians at the oncology and HSCT wards? | - Yes - No |
| Are there daily rounds of the oncologists at the PICU if there is an oncology patient on PICU? | - Yes - No |
| Are there routine meetings between PICU and oncology? | - Yes - No |
| If yes, how frequent are these meetings? | - Daily - Weekly - Monthly - Other (please specify) |
| What are the topics of these meetings? | - Mortality discussions - Complication discussions - Complex-patient discussions - Other, specify…..   (Choose all that apply) |
| In case of an oncology/HSCT patient needs escalation of care, what would most often prompt the oncologist to call the intensivist? | - Increased need for respiratory support - Increased need for cardiac support - Need for RRT - Goals of care discussion - Other, specify….   (Choose all that apply) |
| What in your local hospital would prompt the attending oncologist to admit the patient on PICU? | - Increased need for respiratory support - Increased need for hemodynamic support - Need for RRT - Need for neuromonitoring - Stabilization during goals of care discussion - Other, specify ……   (Choose all that apply) |
| Who/what decides if the child is transferred to the pediatric intensive care unit (PICU)? | - Oncologists - Intensivist - Interdisciplinary decision - Nursing demands - Hospital manager - Logistic settings (as for example negative flow) - Other, specify….   (Choose all that apply) |
| Does your hospital have a palliation unit, consultant or liaison? | - Yes - No |
| If yes, is this service part of the PICU? | - Yes - No, which service if no…… |
| Where does a change of goals of care towards palliation mostly happen? | - At the PICU - At the oncology/HSCT ward - Both at the PICU and oncology/HSCT ward - Outpatient clinic |
| Are PICU consultants involved in goals of care discussions? | - Yes - No - Sometimes |
| Does your hospital have a pain management service/consultant/liaison? | - Yes - No |
| If yes, is this service part of the PICU? | - Yes - No, which service if no….. |
| Does your hospital have a psychological service/consultant/liaison? | - Yes - No |
| If yes, is this service part of the PICU? | - Yes - No, which service if no….. |
| If yes, is psychological support also available for the staff working in the PICU? | - Yes - No - Not applicable |
| **Audit and clinical governance** | |
| Which mechanisms are part of routine practice in your PICU?  (Choose all that apply) | - Local audit - Departmental benchmarking against national standards - Departmental benchmarking against international standards - All PICU admissions of children with an oncological diagnosis are discussed in a joint morbidity/mortality meeting - Other, please specify……. |
|  |  |

**
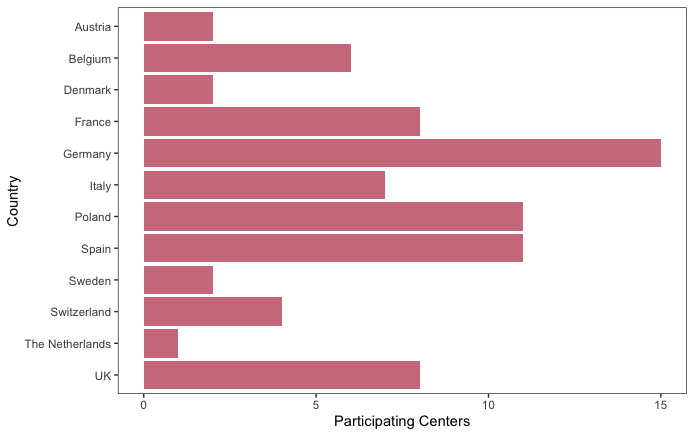
**

**Fig. 1 Pediatric intensive care units participating from around Europe.**

**Table 1. Response rate of PICUs per country**

| **Country** | **Total PICUs connected to pediatric oncology programs** | **Number of responding PICUs** | **Response rate** |
| --- | --- | --- | --- |
| Austria | 4 | 2 | 50% |
| Belgium | 6 | 6 | 100% |
| Denmark | 4 | 2 | 50% |
| France | 37 | 8 | 22% |
| Germany | 60 | 15 | 25% |
| Italy | 18 | 7 | 39% |
| Poland | 25 | 11 | 44% |
| Spain | 36 | 11 | 31% |
| Sweden | 5 | 2 | 40% |
| Switzerland | 8 | 4 | 50% |
| The Netherlands | 1 | 1 | 100% |
| UK | 22 | 8 | 31% |
| Total | 226 | 77 | 34% |
